# Manual Reduction of Incarcerated Abdominal Wall Hernias. A Feasible Option during COVID-19 Pandemic: A Prospective Study

**DOI:** 10.1055/s-0041-1742178

**Published:** 2022-02-01

**Authors:** Konstantinos Bouliaris, Matthaios Efthimiou, Paraskevi Chatzikomnitsa, Christina Kolla, Christos Doudakmanis, Konstantinos Zervas, Anargiros Giaglaras, Georgios D. Koukoulis

**Affiliations:** 1 Department of General Surgery, General Hospital of Larissa, Larissa, Greece

**Keywords:** hernia, visual analog pain score, coronavirus, manual reduction, taxis

## Abstract

**Background** Incarcerated hernia is a common surgical emergency with considerable morbidity or even mortality. Manual reduction (taxis) and elective surgery could be an alternative management approach. This study examines the role of taxis with the adjuvant use of the visual analogue scale (VAS) score in treating incarcerated hernias and thereby decreasing the emergency surgery rate, especially during the novel coronavirus disease 2019 (COVID-19) pandemic.

**Methods** All adult patients admitted to the emergency department of our hospital with incarcerated hernias of anterior abdominal wall were prospectively submitted to hernia manual reduction. The VAS score was used as an adjuvant tool for monitoring the success of this maneuver. Patients with successful taxis and low VAS score were hospitalized for a 24-hour period of observation. On their discharge, they were scheduled for an elective hernia repair. Patients with unsuccessful taxis or with less than a 50% reduction in VAS score after successful taxis were submitted to emergency surgical repair. Age, sex, type of hernias, time until taxis, VAS scores before and after taxis, length of hospital stay, and adverse events for both groups were recorded.

**Results** Between September 2018 and September 2020, 86 patients with incarcerated hernias were included. The types of hernias were incisional in 8 patients, umbilical in 15 patients, inguinal in 56 patients, and femoral in 7 patients. Taxis was successful in 66% of patients with a mean reduction in VAS score from 83 to 17 mm. Following successful taxis, patients were hospitalized for a 24-hour period of observation. No taxis-related complications were observed. Fifty-two patients were safely discharged from hospital and scheduled for an elective repair during the first month. Thirty-four patients were operated emergently. Five patients had successful taxis but with a reduction of posttaxis VAS score less than 50% (a mean reduction from 86 to 62 mm), while taxis failed in twenty-nine patients. Patients with emergency surgery had longer time until reduction and longer stay of hospitalization. In this group, two patients required admission to the intensive care unit while one patient died.

**Conclusion** In this protocolized approach, taxis is a safe and feasible option for most patients with incarcerated hernias. It should be kept in our armament, especially in times when emergency surgery capabilities are under strain like the ongoing COVID-19 pandemic.

Abdominal wall hernia repair is one of the most common surgical procedures with a prevalence ranging from 100 to 500 cases per 100,000 people. 1 Among patients with anterior abdominal wall hernia, 5 to 13% will need emergency surgery due to incarceration. 2 Incarceration is a life-threatening complication and is considered as an absolute indication for emergency surgery because of the risk of occlusion and strangulation of the involved organs. Bowel resection due to intestinal ischemia may be required in 10 to 15% of these cases. 3 Emergency hernia repair rates increase with age, especially in people over 50 years, occur more frequently in males, 4 and result in increased morbidity and mortality by 10 to 20 folds compared with elective surgery. 5 For this reason, elective surgery should be encouraged. In the emergency setting of an incarcerated hernia, the associated surgical morbidity or even mortality could be decreased if another modality could be used safely to reduce incarcerated hernias. Manual reduction of the incarcerated hernia contents with analgesia or/and sedation (also known as taxis), although a matter of debate and not well defined, may be an option. In fact, this strategy could be useful, especially in elderly patients where the associated comorbidities and often antiplatelet or anticoagulant treatment could lead to increased morbidity or might be a contraindication to proceeding directly to surgery. Successful taxis and postponement of an emergency operation until other medical problems have been optimally controlled could provide patients with the benefit of elective surgery. Moreover, concerns about emergency surgery during the ongoing novel coronavirus disease 2019 (COVID-19) pandemic, make the role of taxis a useful adjunct in the management of acute hernias. In these times, hospitals and health care services are overwhelmed. Additionally, patients positive for COVID-19 face risks when undergoing general anesthesia during surgery. The European Hernia Society has produced a recent guidance paper on this subject where taxis is mentioned as an option in cases of incarcerated but not strangulated hernias to allow surgery to be safely delayed for several weeks. 6

The aim of this study was to examine the efficacy and safety of taxis with administration of analgesia in adult patients with incarcerated hernias of the anterior abdominal wall and the use of the visual analogue scale (VAS) pain score as a clinical marker for successful hernia reduction. The validity and simplicity of VAS score has made it among the most commonly used measures of pain intensity in clinical research settings. 7

## Materials and Methods

All adult patients admitted to the emergency department of our hospital with incarcerated hernias of anterior abdominal wall were prospectively submitted to hernia manual reduction. The diagnosis of incarcerated hernia was based on the symptoms of the patient, clinical examination, laboratory tests, and imaging findings. We used the HerniaSurge guidelines definition of incarcerated and strangulated hernia. “Incarceration: inability to reduce the hernia mass into the abdomen” and “strangulation: the blood supply to the herniated tissues is compromised.” 8 The duration of incarceration was defined as the time elapsed from the start of incarceration until hospital arrival. All adult patients with incarcerated hernias of the anterior abdominal wall were included. Exclusion criteria were evidence of strangulation, peritonitis, painless chronic irreducible hernias, and altered mental status. Age, sex, delay in arrival at hospital, type of hernia, past medical history, and presence of coexisting diseases were recorded for each patient. On arrival, the patients were first evaluated by one of the emergency physicians and a surgical resident who took a detailed history and carried out a complete physical examination. When the diagnosis of incarcerated hernia was established, a written consent was obtained and taxis was attempted. VAS pain score (0–100 mm) was used to record the level of pain before and after successful taxis. Tramadol of 50 mg was administered intravenously before attempting taxis. The patient was confined to bed for 15 minutes before the senior surgeon on call attempted taxis by applying steady pressure for approximately 1 to 2 minutes causing only mild discomfort to the patient. Two attempts were allowed with an interval of 15 minutes. Following successful taxis, VAS score was measured at 1 hour and patients were hospitalized for a 24-hour period of observation in case any complications of taxis occurred. On their discharge, patients were scheduled for an elective hernia repair (taxis and elective surgery group). If taxis was unsuccessful or if the patient was still in pain after successful taxis, reduction in VAS score less than 50%, an emergency operation, was carried out (emergency surgery group). In any case, the senior surgeon on call decided which patient should undergo emergency surgery or follow-up and elective surgery. There was a consensus of all senior surgeons to attempt taxis if feasible. For patients underwent successful taxis and elective surgery, follow-up was carried out in the Outpatient Department before the elective surgery, or via phone call in case of distress of the patient between taxis and the date of surgery. After scheduled surgery follow-up was made at 15 days in the Outpatients Department and at 1 month via phone call. Study size was determined by the number of patients who came to the Emergency Department with incarcerated hernias. Mortality and morbidity (Clavien–Dindo classification >II), time until manual reduction, changes in VAS score before and after reduction, as well as average length of hospital stay for both groups, were recorded. All the data were collected by the responsible consultants with the assistance of the on-call residents. All the data were collected in Microsoft Excel v.16 of Microsoft Corporation. Statistical analysis was conducted using IBM Corporation SPSS Statistics v.26. The normality of distribution of the variables was evaluated with the use of Kolmogorov–Smirnov test. All of the variables were normally distributed. Given that the distribution was normal, for nominal variables, the analysis performed using Student's *t* -test, and then Pearson's correlation coefficient was used to assess the strength of the association of the variables. In order to analyze categorical variables, Pearson's Chi-square test was used. Paired *t* -test was used to analyze VAS score before and after taxis. Mean, median, standard deviation, and the frequencies of the variables were calculated with descriptive statistics. Results were expressed as mean ± standard deviation with 95% confidence interval. Statistically significant results were those with *p* < 0.05. We used receiver operator characteristics (ROC) curve to evaluate the use of VAS score as a prognostic factor. We calculated area under the curve as an indicator of the score's prognostic value. Using SPSS Statistics, we calculated the cut-off points, based on the best possible balance between sensitivity and specificity of each value of VAS score. ROC curve was used to evaluate VAS score as an indicator of taxis success and its use in therapeutic decision-making. The study was approved by the ethics committee of the General Hospital of Larissa.

## Results

**Fig. 1:**
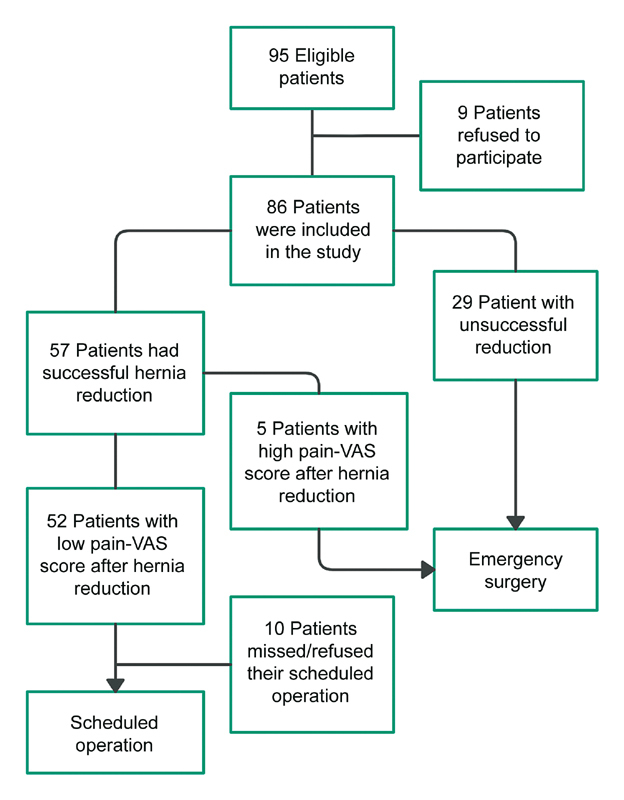
Flow chart of patient admission between September 2018 and September 2020. VAS, visual analog scale.

Between September 2018 and September 2020, 95 consecutive adult patients with incarcerated hernias of the anterior abdominal wall were admitted to the Surgical Ward of our hospital through the Emergency Department. A study flow chart is provided in Fig. 1 . Among the 95 selected patients, 86 were included in the study as 9 patients refused to give a written consent. Patients' types of hernia and outcomes are seen in Tables 1 and 2 . The mean age was 70 years with no difference among the study groups. There were 64 male patients and 22 female patients. The mean delay in arrival at hospital was 18 hours and the mean length of hospital stay was 3.14 days. None of the patients included in the study had a current COVID-19 infection. Fifty-seven patients (66%) underwent successful taxis. Of these, 46 were male and 11 were female patients with a mean age of 69.6 years. The mean VAS pain score was 85 mm before manual reduction and 22 mm after successful taxis while the mean delay in arrival was 15.1 hours.

**Table 1:** Patients' types of hernia and outcomes

Hernia type	Successful reduction	Successful reduction—emergency surgery due to high VAS score	Successful reduction—refusal elective surgery	Unsuccessful reduction—emergency surgery	Total number of patients with reduction attempt
Incisional	5	1	1	3	8
Umbilical	9	–	5	6	15
Inguinal	41	4	3	16	56
Femoral	3	–	1	4	7
Total	57	5	10	29	86

Abbreviation: VAS, visual analog scale.

**Table 2:** Patients demographics, time to reduction, VAS pain score, and days of hospitalization

	SR ( *n* = 52	SRES ( *n* = 5)	*p* -Values SRES compared with SR	URES ( *n* = 29)	*p* -Values URES compared with SR
Age	67.8 years	76.7 years	*p* = 0.2	73.2 years	*p* = 0.08
Sex					
Male	41	5	–	18	*p* = 1
Female	11	–	–	11	
Time to reduction	10.4 hours	34.8 hours	*p* < 0.0001	25.7 hours	*p* = 0.003
P-VAS score					
Before reduction	8.3 cm	8.6 cm	*p* = 0.6	8.6 cm	*p* = 0.3
After reduction	1.7 cm	6.2 cm	*p* < 0.0001	–	–
Days of hospitalization	2.6 days ( *n* = 42)	4.5 days	*p* = 0.04	4.75 days	*p* = 0.0002

Abbreviations: P-VAS, pain-visual analog scale; SR, successful reduction and no emergency surgery; SRES, successful reduction but emergency surgery; URES, unsuccessful reduction and emergency surgery.

In five male (9%) patients from the successful taxis group, emergency surgery was performed due to persistent high VAS pain score. The mean VAS pain score before reduction was 86 mm while after reduction it was 62 mm. These patients had a mean delay in hospital arrival of 34.8 hours and a mean hospitalization of 4.5 days. The diagnosis for these five patients was as follows: one patient with incisional hernia and adhesive small bowel obstruction and four patients with inguinal hernia. Among those four patients, there were two cases of peritoneal sac ischemia, one case of omental torsion and one case of omental ischemia. In the last two cases, adhesions were found between the peritoneal sac and the omentum, despite successful reduction of the contents of the hernia. No enterectomies were performed.

The remaining 52 of 57 (91%) patients were discharged after a 24-hour observation period and were scheduled for an elective hernia repair (taxis and elective surgery group). In this group, the mean age was 67.8 years and the mean delay in arrival and reduction was 10.4 hours. There was also a substantial reduction in the mean VAS pain score from 83 to 17 mm which was statistically significant ( *p* < 0.0001). No taxis-related complications were observed in this group.

Ten patients missed or refused their scheduled operation, while the remaining 42 patients were operated on during the first month with a mean postponement period of 16 days and total mean days of hospitalization 2.6, including the 24-hour posttaxis observation period. No related major postoperative morbidity (Clavien–Dindo classification >II) or mortality were recorded in this group of patients.

In 29 patients (34%), taxis failed, so they underwent emergency surgery. Of these, 18 were male and 11 were female patients. The mean age was 73.2 years, and the mean delay in hospital arrival was 25.7 hours. The mean hospital stay was 4.75 days. Four patients had small bowel ischemia and were submitted to enterectomy. Two patients developed major postoperative complications and required hospitalization in the Intensive Care Unit, while one 95-year-old female patient with femoral hernia died from sepsis 10 days after surgery.

We further investigated the role of taxis as a clinical marker for successful hernia reduction. For that we performed ROC curve for VAS score in four separate conditions. The first one was before taxis, the second was after successful taxis, the third was after successful taxis and yet persistent painful discomfort, and the fourth comprises of patients with unsuccessful taxis that subsequently underwent emergency surgery. Before taxis, AUC was 0.599 which is close to 0.50, meaning that VAS score before taxis has low predictive value. Pretaxis cut-off point was 85 mm (sensitivity = 75% and specificity = 44%). In patients with successful taxis which underwent emergency surgery due to persistent pain, AUC was 0.971 which is very close to 1 and indicates high predictive value. Cut-off point was 55 mm (sensitivity = 75% and specificity = 94%).

## Discussion

Incarcerated abdominal wall hernia is a common abdominal surgical emergency, especially in elderly patients. Reducible hernias are not considered as a surgical emergency. On the other hand, if the hernia is painful and irreducible, the clinical concern should be of possible strangulation of the hernia contents and an emergency surgery is indicated according to recent international guidelines. 8 9 Nevertheless, emergency surgery is associated with significant morbidity or even mortality. In the emergency setting, manual reduction of the hernia contents with analgesia/sedation (taxis) may be an option worth considering. However, the role of taxis is not well defined. In the latest European Hernia Society (EHS) guidelines, although it is not mentioned as a maneuver that may reduce the number of emergency surgeries, no recommendation or statement is made, while in the World Society of Emergency Surgery (WSES) guidelines, it is not mentioned at all. 8 9 There is a relative lack of evidence in the literature regarding this matter and only a few studies have investigated the role of taxis on incarcerated inguinal hernias. As far as we know, there are only two prospective studies. Chen et al examined the role of ultrasound in incarcerated inguinal hernia reduction when manual reduction failed. Also, 112 adult patients with incarcerated inguinal hernia were included in that study. Taxis was successful in 89% of cases. One patient with successful taxis developed sepsis due to bowel necrosis 2 days after reduction. They concluded that although manual reduction is a safe maneuver, the addition of ultrasound guidance can improve the rates of successful taxis and may decrease the rate of emergency surgery. 10 In addition, Harissis et al attempted taxis in 101 patients with anterior abdominal wall incarcerated hernia with a success rate of 60.3%. No taxis-related complication was observed. 11

One drawback of this practice is the risk of reduction en masse which means that the incarcerated bowel is separated from its surrounding structures and reduced into the abdominal cavity but not released from the peritoneal sac. This complication presupposes a constriction at the neck of the peritoneal sac which must be sufficiently mobile within the inguinal canal to permit movement to a new position. Furthermore, the neck must be surrounded by a weak internal ring to which is not attached. 11 12 Under all these circumstances, any forceful manipulation might push the whole of the hernia sac with its irreducible contents through the internal ring into the preperitoneal space. However, the occurrence of reduction en masse seems to be rare. It is reported occasionally and there is no calculated estimation risk for such complication in the medical literature. 12 13 In fact, the risk of reduction of intestine with ischemic/necrotic wall by taxis is very rare. This is due to two reasons. First, only a minority of incarcerated hernias have necrotic tissues (∼10%), 14 and second, the necrotic bowel is not reduced easily, since necrosis occurs in hernias with very narrow orifices. 11

In our series of 86 taxis attempts, we report a success rate of 66%, while no case of reduction en masse was observed. Interestingly, in a recently published review about the role of taxis in incarcerated hernias, the reported success rate was quite similar (70%) and the main factor for unsuccessful reduction was the time lapsed from the onset of pain. The authors reported a linear correlation between the times from the onset of symptoms to strangulation, with the risk of strangulation doubled for every 24 hours of delay. 15 This was shown in our study too. The mean time to reduction was 10.4 hours for the successful taxis and elective surgery group, while in the emergency groups, it was above 25 hours and that difference was statistically significant ( Table 2 ).

It is recommended for patients to remain hospitalized for 12 to 24 hours following taxis to ensure that there are no taxis-related complications. Close clinical observation is mandatory to ensure the success of taxis. One reasonable argument about our protocol could be the attempt of taxis in femoral hernias which is not recommended, as this type of hernias have higher risk for strangulation. 8 However, as it was mentioned above, the necrotic bowel rarely does reduce and taxis usually fails in femoral hernias. This was shown in the study by Harissis et al where among 20 patients with incarcerated femoral hernia, taxis was successful in only 6 patients while the majority needed an emergency surgery. 10 Similarly, in our study only in three out of seven patients with incarcerated femoral hernia taxis were successful with significant pain reduction. These patients were closely observed for 24 hours and on discharge, they were scheduled for timely hernia repair. Pain reduction is a crucial clinical sign for uncomplicated taxis. We used the VAS pain score to evaluate the taxis result. We recorded a significant reduction in VAS pain score in the group of patients with successful taxis and elective surgery in contrast to the patients with clinically apparent reduction of the hernia bulge, where VAS score still remained high leading them to the operating room. The VAS score is a simple and reliable tool in measuring acute abdominal pain and can be used for quantitative assessment and for detecting clinically important changes. 16 It is used mostly for assessing the pain intensity in the Emergency Department or the efficacy of postoperative analgesia. 17 In our study, we used a reduction of VAS score above 50% as a clinical marker for patients' improvement and we estimated that posttaxis VAS score higher than 55 mm indicates that patient may be in need for emergency surgery even after successful hernia reduction. However, due to the small size of our study, it is difficult to suggest a cut-off point in VAS score to use for decision-making. The previous two studies of Chen et al and Harissis et al did not use pain evaluation.

In our study, the benefit of taxis was that over the half of the patients, who would have otherwise undergone emergency surgery, were operated on based on an elective basis with no morbidity or mortality and fewer days of hospitalization compared with the emergency surgery group ( Table 2 ). Morbidity in emergency cases of incarcerated abdominal wall hernias is reported to be between 19 and 30%, and early mortality between 1.6 and 19.4%. 3 Furthermore, in these patients, advanced age (>65 years) and the concurrent increase of comorbid diseases, as well as inadequate preoperative preparations, cause an apparent increase in the rates of mortality and morbidity. 3 4 Hence, the use of taxis can delay surgery until other medical problems are optimally controlled.

In addition, the avoidance of emergency surgery can be very helpful during pandemics such as the ongoing COVID-19. During the severe acute respiratory syndrome-coronavirus-2 (SARS-CoV-2) pandemic, hospital services have undergone major reorganization to increase critical care capacity for patients with coronavirus disease. Subsequently, surgical team members have been disengaged to support wider hospital responses. As a result, in many European countries, emergency surgery capabilities are under strain and vary depending on different levels of outbreak, resource utilization for COVID-19 patients, local testing capacities, and availability of personal protective equipment. During an emergency surgical situation, patients tested negative for COVID-19 may not be truly COVID-19 negative. Therefore, great care should be taken to minimize the risk of infection to health care workers and prevent intrahospital transmission. It is also known that operating on COVID-19-positive patients carries a very high pulmonary complication and mortality rate, 18 and the available data suggest that those with a positive SARS-CoV-2 swab preoperatively should have surgery delayed for at least 4 weeks. 19 Finally, the latest EHS guidelines for the management of adult patients with a hernia during the COVID-19 pandemic suggest, in the emergency setting, taxis under sedation/analgesia followed by observation and the delay of surgery for several weeks as an alternative procedure to emergency surgery. 6

## Limitations

Our study is one of the few prospective studies on manual reduction of abdominal wall hernias and the only one which focuses on the VAS score as a safety net for the success of taxis. However, it does bear certain limitations. First, it is not randomized and second, the number of patients is relatively small. Larger, multicenter controlled studies are needed to further clarify the role of taxis in the treatment of incarcerated hernias.

## Conclusion

Emergency abdominal wall hernia is a common clinical situation, with the time to treatment being of paramount importance to either reduce the hernia content or prevent progression to strangulation or to proceed to surgery, in case of unsuccessful reduction or strangulation is already established. During the COVID-19 pandemic, it is critical to find new ways of treating patients to minimize risks to health care workers. Taxis seems to be a useful and safe tool in treating most patients with incarcerated hernia and therefore decreasing both days of hospitalization and the need for emergency surgery. Time elapsed since the onset of symptoms to manual reduction and the relief of postreduction pain is crucial for the success of this maneuver.
